# Intratumoral bacteria interact with metabolites and genetic alterations in hepatocellular carcinoma

**DOI:** 10.1038/s41392-022-01159-9

**Published:** 2022-09-28

**Authors:** Chen Xue, Junjun Jia, Xinyu Gu, Lin Zhou, Juan Lu, Qiuxian Zheng, Yuanshuai Su, Shusen Zheng, Lanjuan Li

**Affiliations:** 1 State Key Laboratory for Diagnosis and Treatment of Infectious Diseases, National Clinical Research Center for Infectious Diseases, National Medical Center for Infectious Diseases, Collaborative Innovation Center for Diagnosis and Treatment of Infectious Diseases, The First Affiliated Hospital, Zhejiang University School of Medicine, Hangzhou, Zhejiang China; 2 Division of Hepatobiliary and Pancreatic Surgery, Department of Surgery, The First Affiliated Hospital, Zhejiang University School of Medicine, Hangzhou, Zhejiang China

**Keywords:** Gastrointestinal cancer, Prognostic markers

**Dear Editor**,

Hepatocellular carcinoma (HCC), which ranks globally as the third leading cause of cancer-related deaths, is highly prevalent, and most patients are diagnosed with advanced-stage cancer when treatments are largely ineffective.^1^ Thus, there is an urgent need for earlier diagnosis to improve HCC patient outcomes.

Although many types of tumors have intratumor bacteria, the tumor microbiome is poorly characterized because of limitations in the technology for detection. Recently, Nejman et al. found that different tumor types have different microbiomes and that bacterial metabolism is closely associated with clinical features.^2^ They identified cancer type-specific microbial signatures for the microbiome from seven types of human tumors.^2^ Unique microbial reads and signatures were found in tissue and blood within and between most major cancer types for 33 types of cancer in The Cancer Genome Atlas, suggesting that the cancer microbiome might provide novel information for cancer diagnosis.^3^ While better sequencing technology has identified the intratumoral microbiome as an important component of the tumor microenvironment,^4,5^ the microbes, their metabolites, and the underlying gene regulatory network in HCC are poorly characterized.

To study the microbiome, we analyzed 47 pairs of HCC tissues and normal control liver tissues from The First Affiliated Hospital, College of Medicine, Zhejiang University (Supplementary Table. 1). Differences in bacterial communities between samples for core and unique operational taxonomic units (OTUs) were determined by Venn diagram analysis using 16 S rDNA sequencing (Supplementary Fig. 1a). The microbial population structure differed between HCC and control tissues at the phylum, family level, and genus levels (Supplementary Fig. 1b–d). The α diversity of microbes in tumors was analyzed using the Wilcoxon rank-sum test (Fig. 1a), and Bray-Curtis-based principal component analysis (PCA) revealed that the overall microbial composition of HCC tissues deviated markedly from that of normal liver tissues (Fig. 1b). The top 20 microbial taxa with significant differences in relative abundance between cancer and paracancerous tissues are shown in Fig. 1c. We also identified statistically significant differences for representative sequences of the top 100 genera, obtained by multiple sequence alignment, as determined by ANOSIM analysis (analysis of similarities) (Supplementary Fig. 1e, f). The predominant taxa were further characterized by high dimensional class comparisons using linear discriminant analysis (LDA) of effect size (LEfSe) with LDA value distribution histogram and a cladogram shown in Supplementary Fig. 1g, h. Kyoto Encyclopedia of Genes and Genomes (KEGG) analysis identified significant differences in microbial functions for *Oscillospira*, *Mucispirillum*, *Helicobacter*, *Roseburia*, *Ruminococcus*, and *Anaerotruncus* (Supplementary Fig. 1i). Collectively, these results identified the major differences in the microbiomes between HCC and normal liver tissues.

**Fig. 1:**
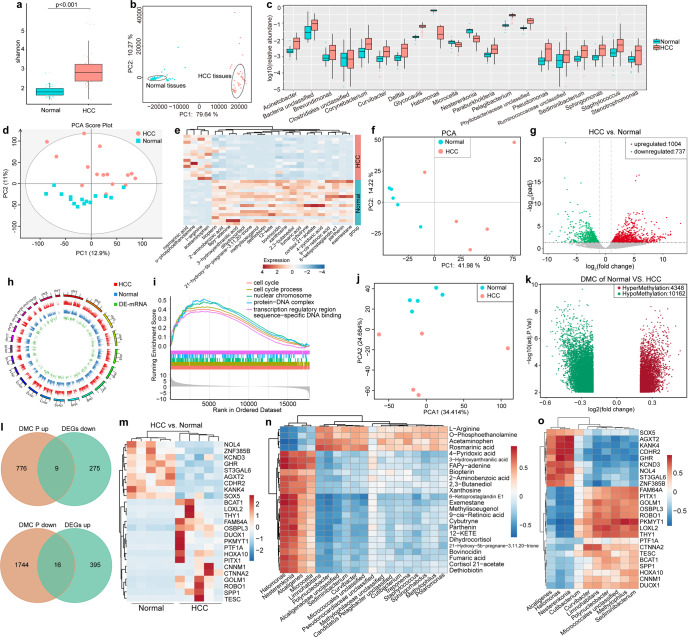
Tumor microbiomes were closely associated with changes in host metabolism and epigenetic and gene expression profiles. **a** Shannon index of the microbiota of samples from tumor tissues and normal control liver tissues. The vertical axis represents the α diversity of the microflora. Different colors represent different groups of samples. **b** PCA scatter plots of microbiota between tissue samples. **c** The top 20 microbial taxa with significant differences in relative abundance between HCC tissues and healthy tissues. **d** PCA scatter plots of metabolomes between tissue samples (*p < 0.05; **p < 0.01). **e** Differential metabolites from cancer tissues and adjacent tissues were identified based on heatmap analysis of Euclidean hierarchical clustering. The horizontal axis represents the different metabolites, the vertical axis represents the samples of different groups, and the red and blue colors represent an increase and a decrease in metabolism, respectively. **f** PCA scatter plots of transcriptomes between tissue samples. **g** Volcano plot of the DEGs in the HCC and paired samples. The abscissa represents the log2FoldChange of gene expression between different groups. The ordinate represents the significance level of the expression difference. **h** Differential gene genome circle diagram. The outermost circle is the chromosome band, the innermost circle is the histogram of the log2FoldChange value of the DEGs, and the middle two circles are the log2(FPKM + 1) distribution diagrams of the two groups of genes. **i** GSEA of DEGs was s**i**gnificantly correlated with cell cycle, cell cycle process, nuclear chromosome, protein-DNA complex, transcription regulatory region, and sequence-specific DNA binding pathway. **j** PCA scatter plots of genes DNA methylation between tissue samples. **k** Volcano plot of the different methylation genes in the HCC and paired samples. The abscissa represents the log2FoldChange of gene expression between different groups. The ordinate represents the significance level of the expression difference. Red dots represent hypermethylation. Green dots represent hypomethylation. Gray dots represent that the difference in methylation was not statistically significant. **l** Venn diagram of gene data showing opposite trends of DNA methylation in promoter regions and corresponding gene expression levels. **m** Heatmap of DNA methylation-related differential genes. **n** Correlation between different bacterial classes and the 24 most abundant metabolites in HCC tissues and healthy liver tissues. **o** Spearman correlation analysis between the microbiome and DNA methylation-related differential genes. Abbreviation: PCA principal component analysis. HCC hepatocellular carcinoma; DEGs differentially expressed genes; FPKM Fragments Per Kilobase per Million

The signature microbial environments in HCC patients suggested that variations in metabolites may be affected by the tumor microbiota. Liquid chromatography-mass spectrometry (LC-MS) identified various metabolites that were used to construct a model based on a plot from PCA and partial least squares discriminate analysis (PLS-DA) which analyzes the correlation between metabolites and samples types. The PCA scores differed markedly in metabolic features between samples (Fig. 1d). Consistent with the results of PLS-DA, a permutation plot confirmed the reliability of the model (Q2 = 0.828, Supplementary Fig. 2a). In addition, the area under the receiver operating characteristic curve (ROC) curve (AUC) was > 0.9 for rosmarinic acid, o-phosphoethanolamine, bioperin, and 2-aminobenzoic acid, calculated by the Random Forest classification model (Supplementary Fig. 2b–e). We identified 214 metabolites by untargeted LC-MS (Supplementary Fig. 2f), and a heatmap of the top 24 differential metabolites (False Discovery Rate < 0.05, |log2(Fold Change)| > 1) showed that acetaminophen, L-arginine, O-phosphoethanolamine, and Rosmarinic acid were highly enriched in HCC tissues compared with the control, while Exemestane, Parthenin, 6-ketoprostaglandin E1, N9-cis-retinoic acid, 4-pyridoxic acid, and Cortisol 21-acetate were less abundant in tumor tissues (Fig. 1e). In addition, KEGG analysis identified several metabolic pathways with significant differences such as ABC transporters, Purine metabolism, and Vitamin digestion and Absorption (Supplementary Fig. 2g). Thus, the metabolic patterns were distinct for HCC tissues compared to normal liver tissues.

Because there is mounting evidence that the microbiome affects host epigenetic regulation, we analyzed the effect of DNA methylation on differential gene expression by determining the transcriptome and the epigenome for five pairs of HCC tumor and normal liver tissues. The differences in the expression of genes between the two tissue types are shown by PCA (Fig. 1f), and the distribution of transcript levels in different samples was determined from the number of reads for all transcripts in each sample (Supplementary Fig. 3a). RNA-seq showed that 737 transcripts were markedly downregulated and 1004 were upregulated in HCC tissues (Fig. 1g). The differentially expressed genes (DEGs) are shown as a heatmap (Supplementary Fig. 3b) with their distribution on 22 chromosomes (Fig. 1h). Gene set enrichment analysis (GSEA) for these genes revealed that highly expressed genes were mainly enriched in the cell cycle, cell cycle process, nuclear chromosome, protein-DNA complexes, and similar pathways (Fig. 1i). The KEGG and Gene Ontology (GO) enrichment analysis for the DEGs is shown in Supplementary Fig. 3c–f. To further understand the function of the DEGs, we constructed a protein-protein interaction network using the STRING database (https://cn.string-db.org/) (Supplementary Fig. 3g). We further determined the DNA methylation profiles for five pairs of HCC tissues and normal liver tissues. The distribution of methylation sites for all samples (*β* value ≤ 0.2 is an unmethylated site, a β value ≥ 0.6 is a methylated site, and values between these two indicate intermediate methylation) is provided in supplementary Fig. 4a. The methylation patterns of these DEGs were distinct between the HCC and normal tissues (Fig. 1j). Through unsupervised PCA, the holistic quality of the data for tumor sand controls was determined (supplementary Fig. 4b). Using probes corresponding to the hg19 reference genome position, we searched for differential methylation sites at the same position in the genomes of multiple samples. The volcano plot for differential methylation sites between samples is shown in Fig. 1k and a differential methylation scatter plot is shown in supplementary Fig. 4c. The methylation of different regions of the genome results in different regulatory mechanisms for gene expression. Therefore, we determined the distribution of differentially methylated cytosines (DMC) in the genome (Supplementary Fig. 4d) using cluster analysis for the first 500 significantly different methylation sites (Supplementary Fig. 4e). The functional enrichment results for differentially methylated genes are shown in Supplementary Fig. 5a–d. Increased DNA methylation of CpG islands within the promoter regions of genes suppressed transcriptional initiation and thereby silenced these genes. Conversely, decreased DNA methylation of promoter regions led to increased expression of target genes. We identified 25 DNA methylation-related DEGs (Fig. 1l), including *NOL4*, *ZNF385B*, *KCND3*, *GHR*, *ST3GAL6*, *AGXT2*, *CDHR2*, *KANK4*, *SOX5*, *BCAT1*, *LOXL2*, *THY1*, *FAM64A*, *OSBPL3*, *DUOX1*, *PKMYT1*, *PTF1A*, *HOXA10*, *PITX1*, *CNNM1*, *CTNNA2*, *GOLM1*, *ROBO1*, *SPP1*, and *TESC* (Fig. 1m).

Using Spearman correlation analysis we found that some microbes, for example, *Halomonas*, were significantly positively associated with some metabolites but negatively correlated with L-Arginine, O-Phosphoethanolamine, Acetaminophen, and Rosmarinic acid (Fig. 1n and Supplementary Fig. 6a). We analyzed the microbiome and host transcriptome interactions by Spearman correlation and found that 10 metabolome-related microbial taxa were closely associated with 25 methylation-related DEGs (Fig. 1o and Supplementary Fig. 6b). For example, Alcaligenes correlated positively with *SOX5*, *AGXT2*, *ST3GAL6*, *KANK4*, and *ST3GAL6*, but correlated negatively with *PITX1*, *GOLM1*, *OSBPL3*, and *PKMYT1*.

The microbiome, metabolome, host transcriptome, and DNA methylation of HCC tissues and paired normal tissues revealed several intratumoral microbial signatures. Furthermore, the correlation between microbial species and metabolites, DNA methylation and gene alterations, and microbial species and gene alterations may provide a better understanding of the tumor microenvironment. Microorganisms closely associated with the occurrence and progression of tumors may serve as novel biomarkers for the diagnosis and prognosis of patients with HCC. However, further validation of the presence of bacteria in HCC tumors and a demonstration of their effect on the phenotypes of tumor cells are still needed.

## Supplementary information

SUPPLEMENTAL MATERIAL

## Data Availability

The datasets generated during and/or analysed during the current study are available from the corresponding author on reasonable request. And the RNA sequence data have been deposited to the BioProject database (BioProject ID: PRJNA862293). Project information will be accessible with the link http://www.ncbi.nlm.nih.gov/bioproject/862293.
