# Supplementary material for: Intratumoral bacteria interact with metabolites and genetic alterations in hepatocellular carcinoma
Source: Signal Transduct Target Ther. 2022 Sep 28;7:335. doi: 10.1038/s41392-022-01159-9 (PMC9515207; doi:10.1038/s41392-022-01159-9)
Supplement: Supplementary file 1 — SUPPLEMENTAL MATERIAL [file 41392_2022_1159_MOESM1_ESM.pdf]

Supplemental Material and Figures for

Intratumoral bacteria interact with metabolites and genetic alterations in hepatocellular carcinoma

Chen Xue[1#], Junjun Jia[2#], Xinyu Gu[1#], Lin Zhou[2], Juan Lu[1], Qiuxian Zheng[1], Yuanshuai Su[1], Shusen Zheng[2*], Lanjuan Li[1*]

[1] State Key Laboratory for Diagnosis and Treatment of Infectious Diseases, National Clinical Research Center for Infectious Diseases, National Medical Center for Infectious Diseases, Collaborative Innovation Center for Diagnosis and Treatment of Infectious Diseases, The First Affiliated Hospital, Zhejiang University School of Medicine, Hangzhou, Zhejiang, China.

[2] Division of Hepatobiliary and Pancreatic Surgery, Department of Surgery, The First Affiliated Hospital, Zhejiang University School of Medicine, Hangzhou, Zhejiang, China.

[#]These authors contributed equally: Chen Xue, Junjun Jia, and Xinyu Gu

***Corresponding author**

Lanjuan Li

State Key Laboratory for Diagnosis and Treatment of Infectious Diseases, The First Affiliated Hospital, College of Medicine, Zhejiang University, No. 79 Qingchun Road, Shangcheng District, Hangzhou, Zhejiang 310003, China. Tel: 86 0571-87236459; Email: ljli@zju.edu.cn (Lanjuan Li)

Shusen Zheng

Division of Hepatobiliary and Pancreatic Surgery, Department of Surgery, The First Affiliated Hospital, College of Medicine, Zhejiang University, Hangzhou, Zhejiang, China. Tel: 86-571-87236466, shusenzheng@zju.edu.cn (Shusen Zheng).

This file includes:

Materials and Methods

Figures legends of Fig. S1 to Fig. S6

Supplementary table 1

References

Materials and Methods

Patient recruitment and tissues sample collection

Surgical tissues were collected from HCC patients without preoperative therapy in the First Affiliated Hospital, College of Medicine, Zhejiang University. A total of 47 pairs of HCC tissues and adjacent normal tissues were collected with the consent of patients. Cancerous tumors and adjacent tissues were cut into 3–5 pieces of ~0.5 cm diameter stored in liquid nitrogen. We collected basic patient information, tissue collection time, and tumor size. All experiments were approved by The First Affiliated Hospital, Zhejiang University School of Medicine (IIT20210168B-R1).

High-throughput 16S ribosomal DNA (rDNA) sequencing and analysis

DNA was extracted using the DNA-sorb B kit (NextBio, Russia). The 16S rDNA gene sequencing was performed using the Illumina MiSeq platform.[1] The interleaving of the raw data was removed and sequences were spliced. Bacterial richness and diversity were analyzed by sampling-based operational taxonomic unit (OTUs) analysis. Alpha diversity was used to calculate the complexity of species diversity for each sample. Beta diversity assessed through principal component analysis (PCA) by the R package (http://www.R-project.org/) was used to determine the distribution of microbial communities. Bacterial taxonomic analyses and bacterial phylum and genus comparisons for tumor and normal liver tissues were determined by the Wilcoxon rank-sum test. The differentially abundant taxa were evaluated by LDA coupled with an effect size method (http://huttenhower.sph.harvard.edu/lefse/).

Liquid chromatography-mass spectrometry (LC-MS) -based metabolomics data acquisition and analysis

After washing 50 mg of tumor tissue and normal liver tissues with saline and drying, the sample was homogenized in three volumes of normal saline for one min. Then 1ml tissue extract (75% 9:1 methanol: chloroform, 25% H2O) (-20 °C) and 3 steel beads were added in samples and the mixture was vortexed for 2 mins. Ultrasound at room temperature for 30 minutes and incubated at 4℃ for 30 min, centrifuged at 12,000 rpm at 4°C for 10 min, and 200 μL of the supernatant was transferred to a new

microcentrifuge tube and samples were freeze-dried in vacuum; Then 200 μL 2-chlorobenzalanine (4 ppm) 50% acetonitrile solution was added and vortexed for 30 s. The sample was used for further LC/MS analysis by Thermo Q Exactive HF-X.

The original data were converted into NetCDF format using the Agilent MSD workstation software, and the linear pattern recognition technique was used. After centralization and standardization, a three-dimensional model was imported into SIMCA-P1 1.5 software (Umetrics, Umea, Sweden). To observe sample aggregation, dispersion, outliers, PCA, and Partial Least Squares-Discriminant Analysis (PLS-DA), was performed for data processing. To verify the multidimensional statistical results, the Mann-Whitney U test was used to analyze the different metabolites.

RNA-seq

RNA was extracted from frozen tissues using TRIzol reagent (Invitrogen Life Technologies, Waltham MA, USA) and RNA concentration and purity were determined by NanoDrop. After inspection of RNA-seq library quality, the different libraries were pooled according to the requirements for effective concentration and target data volume and then sequenced on Illumina Novaseq 6000 using fluorescently labeled dNTPs, DNA polymerases, and adapter primers in the sequencing flow cell.

Raw sequencing data were analyzed as follows: 1. Sequencing data quality evaluation: RNA-seq libraries were evaluated through the statistics of sequencing error rate, data volume, alignment rate, etc. A follow-up analysis was carried out if the standard was met, otherwise, the libraries were rebuilt or additional tested. 2. Information mining and analysis: The standard analysis process of RNA-seq included quality control, comparison, quantification, significant difference analysis, and function enrichment analysis.

Illumina human methylation 850K microarray and data analysis

Total DNA was extracted from HCC tissues and paired liver tissues using MagAttract M48 DNA Manual Kit (Qiagen, Germany). DNA was quantified using NanoDrop 2000c (Thermo, USA). Illumina Human Methylation 850K microarray profiling was performed to determine the DNA methylation of the samples. The differential methylation analysis of genes between paired tissues mainly included the following

steps: DNA bisulfite-conversion, DNA denaturation amplification, DNA fragmentation, precipitation, resuspension, and the Infinium Methylation EPIC methylation chip hybridization. Methylation microarray data were analyzed using the Bioconductor workflow including wateRmelon, methylumi, ChAMP, minfi, missMethyl, and other R packages.[2]

Statistical analyses

All data analyses were conducted by SPSS 22.0 software (SPSS Inc, USA). The differences between the two groups were analyzed by Student's t-test. Transcriptome integration with DNA methylation profiles was performed by differential methylation analysis of all probes selected to cover DEGs. Spearman correlation analysis was used to analyze the relationship between the microbiome and metabolomics, and microbiome and DNA methylation-related DEGs. Values are expressed as the mean ± standard deviation. A p-value < 0.05 was considered statistically significant.

Figures

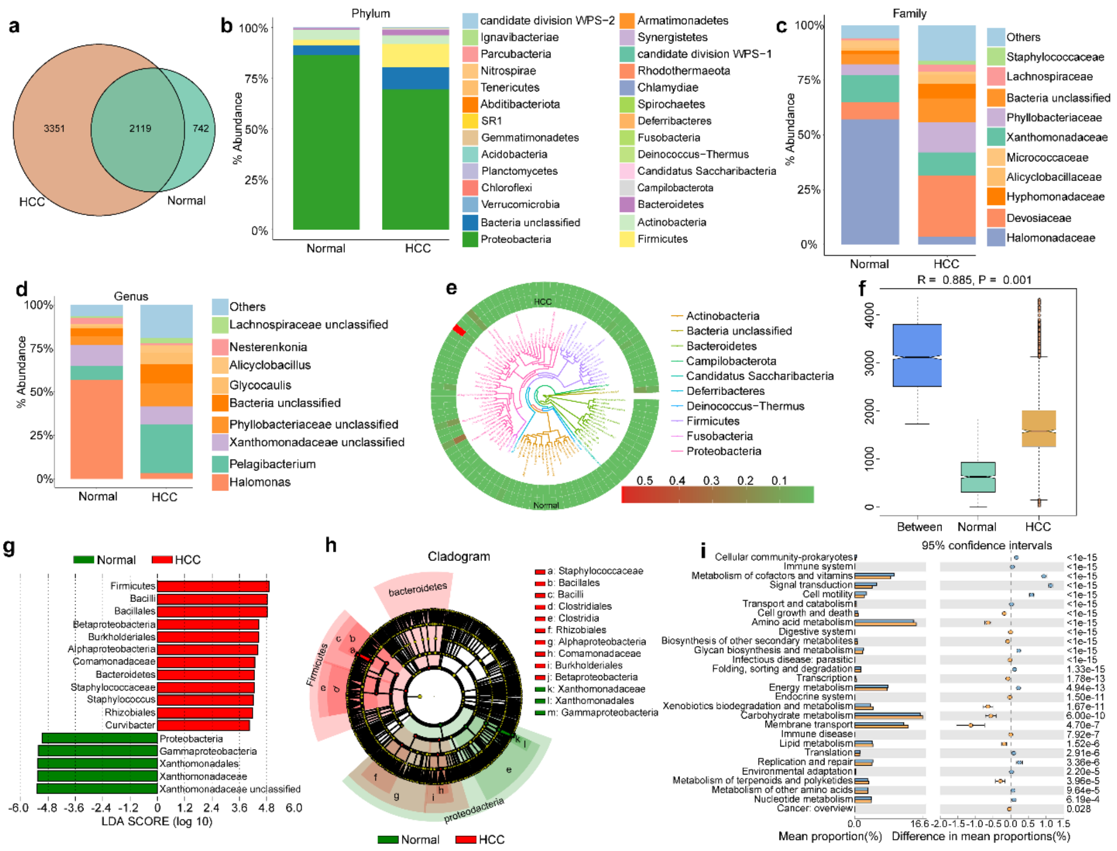

Supplementary Fig. 1 Differences in microbial composition in HCC tissues and adjacent tissues. **a**. Venn diagram of OTUs. **b–d**. The 10 most abundant taxa at phylum, family, and genus level. **e.** Phylogenetic tree constructed from representative sequences of species at the genus level. The colors of the branches and sectors represent their corresponding gates, and the heatmap outside the sector ring represents the distribution in the abundance of the genus in different samples. **f**. The boxplots for differences between groups and within groups using ANOSIM analysis. **g–h**. Differentially abundant taxa between HCC tissue and adjacent tissue analyzed by LEfSe are shown as a histogram (g) and a taxonomic cladogram (h). **i.** Comparison of differences in the abundance of KEGG-pathways in HCC tissues and adjacent tissues. Abbreviation: HCC, hepatocellular carcinoma; OTUs, operational taxonomic units; LEfSe, linear discriminant analysis of effect size. KEGG, Kyoto Encyclopedia of Genes and Genomes.

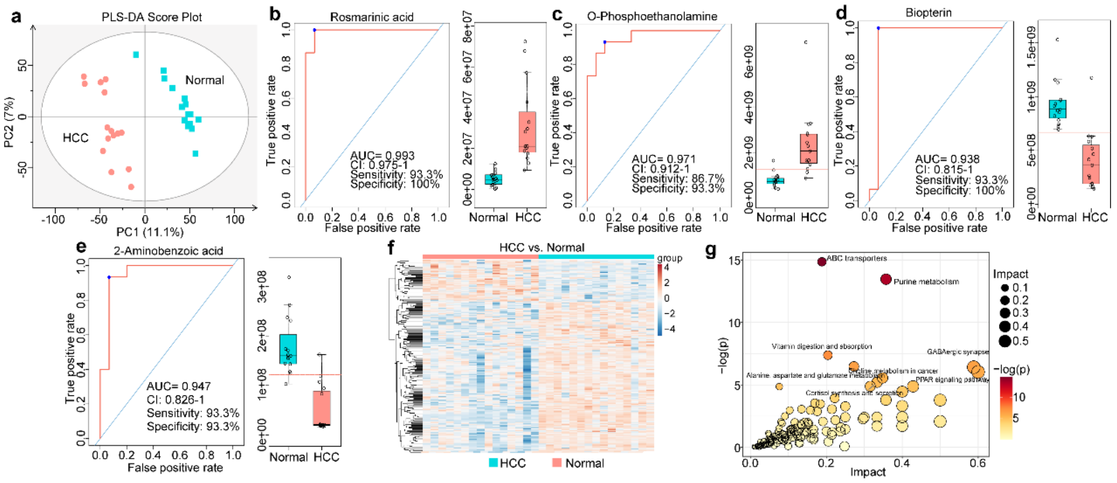

Supplementary Fig. 2 Differences in metabolites in HCC tissues and normal liver tissues. **a**. PLS-DA scores plot of the metabolome between HCC tissues (red) and adjacent normal tissues (green). **b–e**. The differences in metabolites between HCC tissues and normal liver tissues including Rosmarinic acid (b), O-phosphoethanolamine (c), Bioperin (d), and 2-aminobenzoic acid (e) were assessed by ROC curves. **f**. Cluster heatmap of differential metabolites. **g**. Differential KEGG metabolite pathway analysis. Abbreviation: OPLS-DA, orthogonal projections to latent structures discriminant analysis; KEGG, Kyoto Encyclopedia of Genes and Genomes; ROC, Receiver operating characteristic.

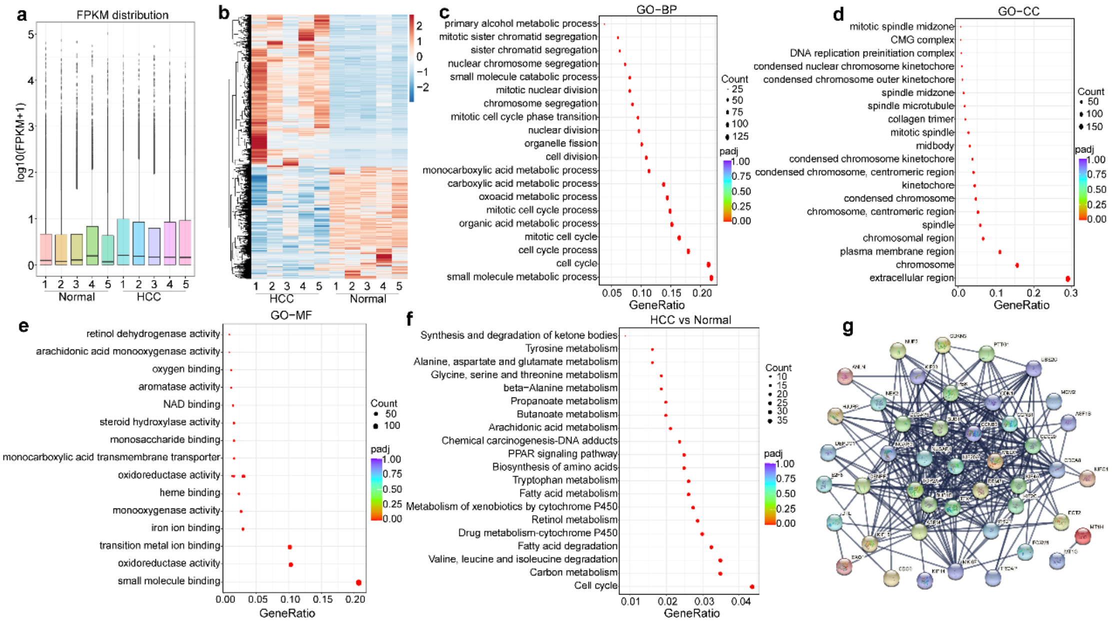

Supplementary Fig. 3 Transcriptome expression difference between HCC tissues and normal liver tissues. **a**. Box plots show the distribution of transcript expression levels

in different samples. **b**. Cluster heatmap of DEGs. **c**–**e**. GO functional analysis of DEGs: BP annotation map of DEGs (c), CC annotation map of screened DEGs (d), and MF annotation map of screened DEGs (e). **f**. KEGG annotation diagram of screened DEGs. **g**. The protein-protein interaction of DEGs. Abbreviation: HCC, hepatocellular carcinoma; DEGs, differentially expressed genes; BP, biological process; CC, cellular component; MF, molecular; KEGG, Kyoto Encyclopedia of Genes and Genomes; GO, Gene Ontology.

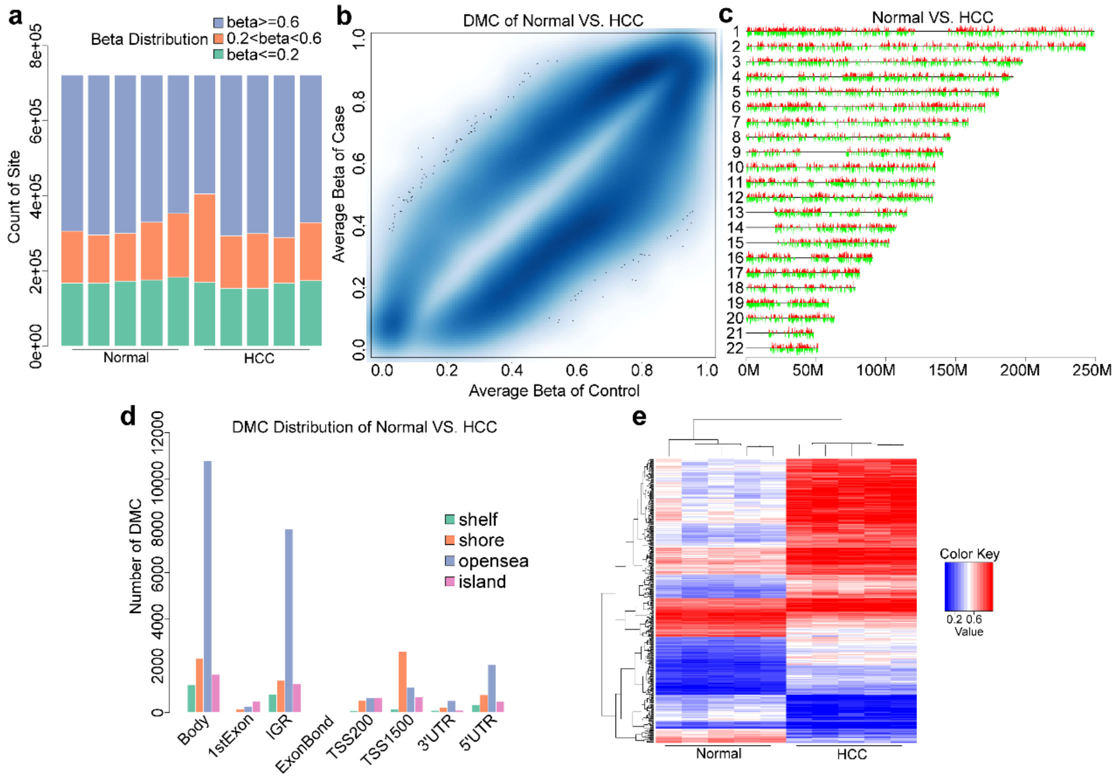

Supplementary Fig. 4 The methylation profile of HCC tissues and normal tissues. **a**. Distribution of beta values after normalization. **b**. Scatter plot of DMC. **c**. The location distribution of DMC on chromosomes. **d**. Distribution of DMC in genomic regions. **e**. Heatmap of DMC between different samples. Abbreviation: HCC, hepatocellular carcinoma; DMC, differentially methylated cytosines.

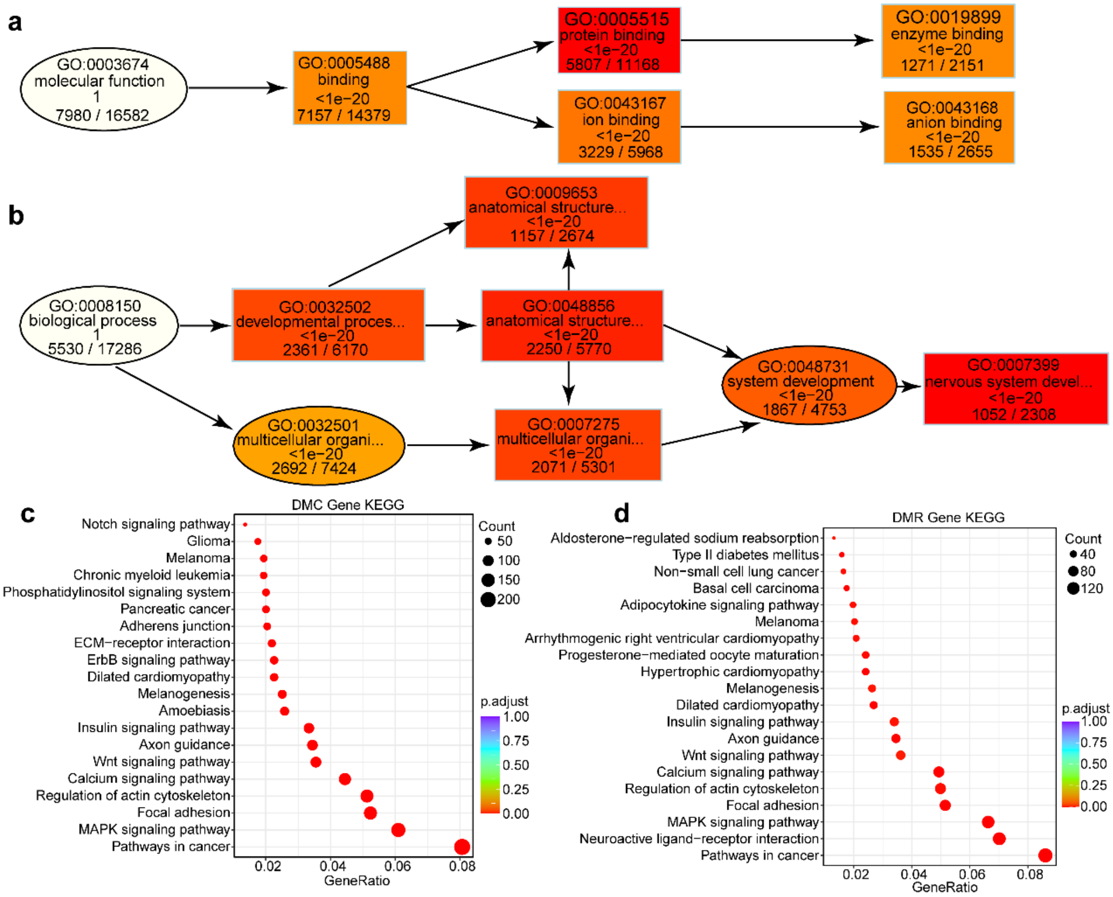

Supplementary Fig. 5 The functional enrichment analysis results for differentially methylated genes. **a**. DAG plot of GO enrichment analysis for DMC corresponding genes. **b**. DAG plot of GO enrichment analysis for DMR corresponding genes. **c**. KEGG enrichment analysis of DMC corresponding genes. **d**. KEGG enrichment analysis of DMR corresponding genes. Abbreviation: DAG, Directed Acyclic Graph; DMR, differentially methylated region; DMC, differentially methylated cytosines; KEGG, Kyoto Encyclopedia of Genes and Genomes; GO, Gene Ontology.

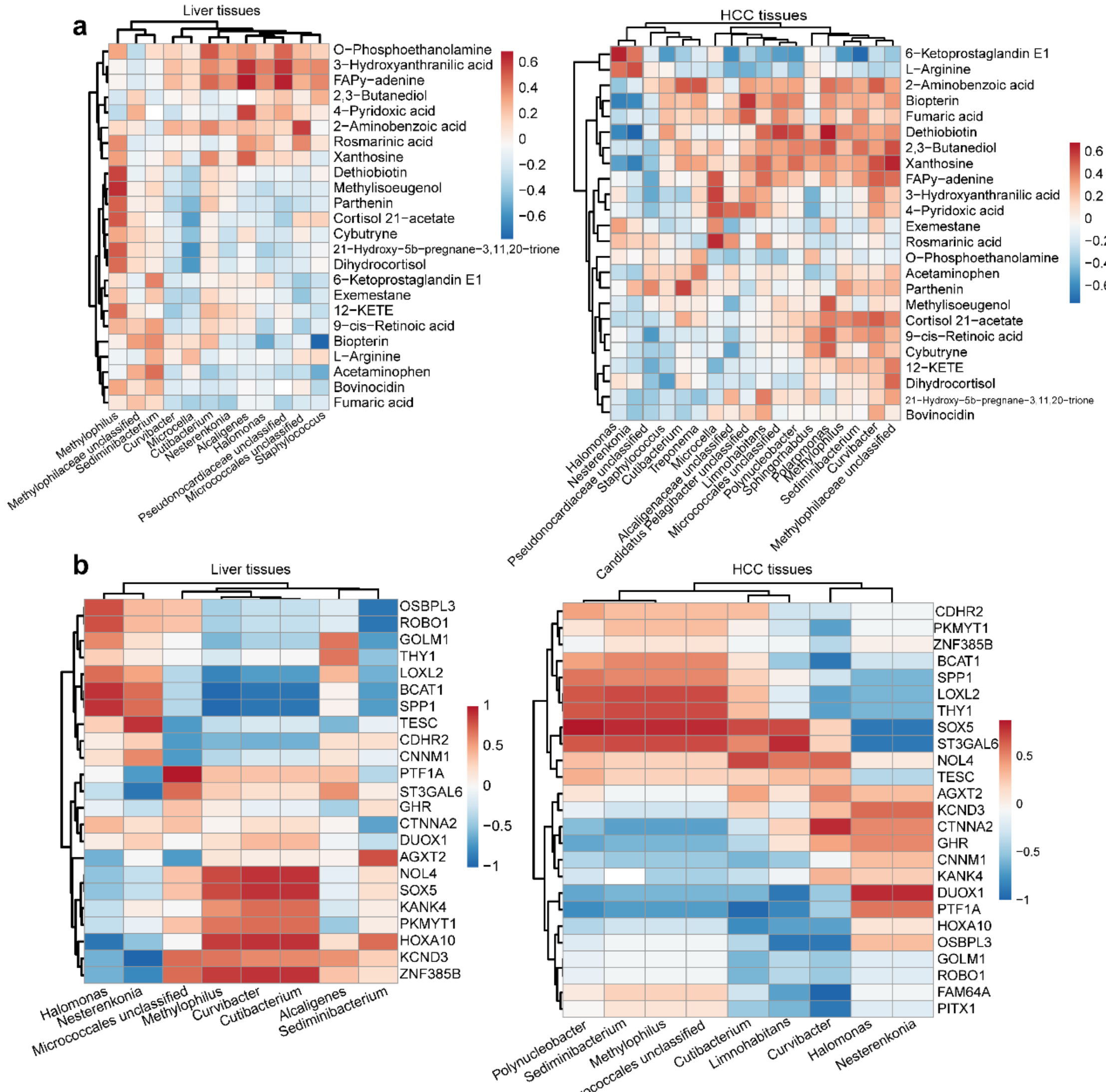

Supplementary Fig. 6 The correlation between microbial species and metabolites, and microbial species and host transcriptome. **a.** Correlation between different bacterial classes and the 24 most abundant metabolites in liver tissues and HCC tissues, respectively. **b.** Spearman correlation analysis between the microbiome and DNA methylation-related differential genes in liver tissues and HCC tissues, respectively.

Supplementary Table 1 The clinicopathological features of 47 patients of hepatocellular carcinoma

Features	Patient cohort (n = 47)
Age (mean ± SD)	56.94±9.92
Gender (male%)	42 (89.36%)
BMI (kg/m^2)	22.38±3.57
Tumor size	
<5cm	18 (38.30%)

>=5cm	29 (61.70%)
Grade	
G1-G2	29 (61.70%)
>G2	18 (38.30%)
AFP (ng/mL)	
<20	24 (51.06%)
>=20	23 (48.94%)

REFERENCES

1. Fu A. et al. Tumor-resident intracellular microbiota promotes metastatic colonization in breast cancer. *Cell.* **185**, 1356-1372.e26 (2022).
2. Maksimovic J. et al. A cross-package Bioconductor workflow for analysing methylation array data. *F1000Res*. **5**, 1281 (2016).
